# Modern Sociology

**Published:** 1903-04-18

**Authors:** 


### Modern Sociology.

### RESIDENTIAL CLUBS FOR CLERKS.

Over five thousand shares have already been taken in the company called "Ingram Houses," formed for the purpose of providing better accommodation for the city clerk —the young man in bank or insurance office, with a salary of something like 30s. or £2 a week. The plan in question is the result of investigations undertaken by the Church of England Men's Society, and the proposed houses are to be called after the Bishop of London, who, however, makes it clear that he is not concerned with the details of the idea.

At a meeting held some months ago the Bishop spoke on the need for some scheme comprising the advantages of a residential club. He said that the lives of many young men in London to-day were terribly lonely. Accustomed as they were, at home or in the country, to the advantages of social life, they found themselves in London cut off from all such intercourse, and condemned to "return at night to their solitary diggings, humanly speaking, to be left alone for the evening." He said he had known many who had taken to bad ways as an escape. The moral temptations of such a life were very great. "You can hardly imagine," one of his young friends had said to him, "what it is, in the midst of all the temptations, to have to say No, No, No, every night." The Bishop added that the lack of opportunity for physical exercise was another evil, and that the life in question was a cramped one.

Having detailed the need of something better, he proceeded to say how he thought it might be met. He would have something on the model of the Rowton Houses; the cost must be moderate, for the clerk had to live on "precious little." He should have two rooms, perhaps a swimming bath, and opportunities for social intercourse with others in the same line of life as himself. Debates, the Bishop said, were very good things; they sharpened the intellect, even if the House of Lords and the Church of England were incidentally abolished.

There should be no religious test, no clergyman at the head (this was his own idea), but a "breeze of good strong public opinion" should pervade the place, and there should be something in the atmosphere which would encourage the residents to lead a spiritual life in connection with their church or chapel. From such a club he looked for great things, and he stated that he could put his hand at the moment on 500 young men of another class, who were sober and self-respecting owing to one Working Boys' Club. Someone had said this was no matter of charity; he would be ashamed of the men they were catering for if such an idea were possible. Financially, he believed such a scheme would bring in a good dividend, but a better one still would be found in the improvement in the lives of the young men they wanted to help.

The scheme of "Ingram Houses," now well established as a joint-stock company, is somewhat as follows: A site has been acquired in the Stockwell Road, two minutes from the City and South London Electric Railway, and on the direct route, by bicycle, for the country in the Surrey direction. The plans of the building which it is proposed to put up have been prepared by Mr. Arthur T. Bolton, A.R.I.B.A., and are very attractive. The general outside view is of a block six or seven storeys high, the shape being that of an elongated St. Andrew's cross. This ensures air on all sides, and apparently the hollows might be filled by gardens. Gardens, a lodge, bicycle house, etc., occupy the front facing Stockwell Road, and there is a private approach, the whole effect being rather like that of a college planted in the midst of small houses. On the lower ground floor are the various kitchen and service offices, lecture-hall, billiard-room, bath-room, lavatories, lockers and box-rooms, a carpenter's shop, and lift, telephone, etc. On the ground floor half the centre and one of the wings are taken up by the dining-room, which is to seat 200; there is a second billiard-room (each has three tables), a reading and writing-room, and a smoking-room. The floors above are all occupied by the bedrooms, in double rows on either side of a passage extending along the four wings. There are bath-rooms also on the bedroom floors, and a fire-escape is provided. The perspective sketches of lecture and dining-halls show very artistic rooms with wainscotted and small-paned windows; the ceiling of the lecture hall is supported by pillars.

The size of the bedrooms, which came in for some criticism at the meeting, is not less than 7 feet by 10 feet; the price ranges from 7s. for those on the top floor; the corner rooms, and those on each succeeding lower floor, being 1s. extra; the lowest are considered the most desirable. The maximum rent is 12s. The house is designed for 250 persons, and the large staff will include a good cook (a very important point), 6 men-servants and 22 housemaids.

It is to be self-supporting, and it is estimated that a dividend of perhaps 4 or 5 per cent. may be paid to shareholders. The return from the weekly rents of 250 men would be £6,000 a year, and the yearly cost at least £3,480. The cost of putting up the building is estimated at £42,800, and £6,000 is allowed for furnishing, in addition to £10,826 (less a mortgage of £6,600) paid for the site, making a total of £53,026.

In the discussion which followed the setting forth of the scheme at the meeting referred to above, some of the speakers seemed to fear interference with the liberty of the residents, while others were afraid moral influence would not be exerted as they thought it should be. Again, one gentleman deprecated the large number of rooms, while others thought it would be found, as it had been in the case of the Rowton Houses, that safety, from a financial point of view, lay in larger numbers still. One thought the rooms too highly priced for the clerk with a small salary; and another wanted to know whether personal recommendations would be required. To this it was answered that the only method that had been thought of was that directors of business houses might perhaps be disposed to subscribe £250 in shares, in respect of which one bedroom would be reserved for an employé, while the employers would have the privilege of nominating the resident. A great deal it was added, would lie in the hands of the manager of the club. To the question as to whether the members would have any share in the management, it was answered that this would be a question of internal economy, and that anything the members suggested would be considered.

One anxious gentleman was afraid the members might be rowdy, and then who was going to turn them out?

Although it is anticipated that a dividend will be paid to shareholders (the sum available for the purpose is estimated at £2,670 yearly), Ingram Houses are not to be looked upon as a paying concern. We understand that the profit over and above that amount will probably be put to a reserve fund, and that the idea is to extend the scheme and in time build other houses of the same character. There is no doubt whatever that the scheme is an excellent one, and that it will prove a Godsend to many lonely clerks. Lord Rowley believes it will meet with social and financial success. It is backed up by a number of notable persons, including the Bishop of London and Canon Scott Holland, Mr. W. R. Hoare (chairman), and Messrs. C. J. Barrington Hurst, G. A. King and W. F. Richmond. The registered offices are at 37 Norfolk Street, W.C.

